# Asymptomatic thyroiditis presenting as pyrexia of unknown origin: a case report

**DOI:** 10.1186/s13256-018-1590-6

**Published:** 2018-02-23

**Authors:** Chamara Dalugama

**Affiliations:** Department of Medicine, University of Peradeniya, Peradeniya, Sri Lanka

**Keywords:** Sub-acute thyroiditis, Pyrexia of unknown origin, Asymptomatic, Management

## Abstract

**Background:**

Pyrexia of unknown origin is a difficult and challenging problem for the physician. Endocrine disorders, such as subacute thyroiditis, rarely present with pyrexia of unknown origin. Subacute thyroiditis can have a broad spectrum of clinical presentations including fever and biochemical thyrotoxicosis without overt signs or symptoms.

**Case presentation:**

A previously healthy 42-year-old Sri Lankan Sinhalese man was extensively investigated for a prolonged fever of 3 weeks with high inflammatory markers. He had mild tenderness over his neck with cervical lymphadenopathy with no thyrotoxic symptoms or signs. An ultrasound scan revealed an enlarged thyroid with increased vascularity and he had suppressed thyroid-stimulating hormone with elevated free thyroxine and free triiodothyronine hormone levels. Fine-needle aspiration cytology confirmed thyroiditis. He responded well to low-dose steroids.

**Conclusion:**

Subacute thyroiditis should be considered in the diagnostic workup of pyrexia of unknown origin even in the absence of overt toxic symptoms of thyroid hormone excess.

## Background

Pyrexia of unknown origin (PUO) is a diagnostic challenge for the treating physician. Subacute thyroiditis rarely presents with PUO. Subacute thyroiditis is a rare self-limiting inflammatory condition probably viral in origin with a genetic predisposition. It commonly presents with a painful swelling of the neck and mild thyrotoxic symptoms with raised inflammatory markers and rarely with other atypical clinical features such as lymphadenopathy. Thyroid antibodies are commonly negative. Management includes nonsteroidal anti-inflammatory drugs (NSAIDs) or steroids. We report the case of a Sri Lankan Sinhalese man presenting with a fever for 3 weeks without neck pain or thyrotoxic symptoms diagnosed as having subacute thyroiditis and his recovery following a course of low-dose steroids.

## Case presentation

We report a case of a 42-year-old Sri Lankan Sinhalese man who presented with fever of 3 weeks’ duration. He did not smoke tobacco or consume alcohol; he was an executive officer in a company. He had daily fever spikes with generalized malaise. He complained of severe loss of appetite and weight loss of 5 kg over the 3 weeks. He denied having cough, alteration of bowel habits, or urinary symptoms. He did not have past history or contact history of tuberculosis.

On examination he was febrile. He had mild pallor, but not icteric. He had bilateral tender cervical lymphadenopathy the largest measuring 1 cm with mild tenderness over the anterior neck without an obvious swelling suggestive of goiter. His pulse rate was 72 beats per minute with a blood pressure of 120/80 mmHg and the rest of the cardiovascular system examination was normal. Respiratory and abdominal examinations were unremarkable with normal neurological findings.

A complete blood count showed hemoglobin of 10.7 g/dL, white count of 9.1 × 10^6^/microL and platelet count of 350 × 10^3^/microL. His erythrocyte sedimentation rate (ESR) was 80 mm in the first hour and C-reactive protein (CRP) was 112 mg/L. Blood film showed normochromic normocytic cells with moderate rouleaux formation. His serum albumin was 46 g/L with normal levels of transaminases and bilirubin. Renal functions were within the normal limit. Three sets of blood cultures were sterile. In two-dimensional echocardiogram, the valves and the endocardium were free of vegetations. An ultrasound scan of his neck revealed diffusely enlarged thyroid gland with increased vascularity. There were multiple lymph nodes with preserved architecture; the largest measuring 1 cm. Thyroid profile showed thyroid-stimulating hormone (TSH) of 0.012 MIU/mL (normal range 0.27 to 4.7 MIU/mL), free thyroxine of 42.08 pmol/L (normal range 10.5 to 19.4 pmol/L), and free triiodothyronine 8.71 pmol/L (normal range 4.0 to 8.3 pmol/L). Fine-needle aspiration cytology of the thyroid showed evidence of thyroiditis with clustered epithelioid cells, scattered lymphocytes, and a few multinucleated giant cells. Facilities for radio-iodine imaging were not available during the time of investigations. Thyroid peroxidase antibodies were negative. A diagnosis of subacute thyroiditis was made. He was clinically completely euthyroid with no clinical features of hyperthyroidism such as palpitations, heat intolerance, and frequency of stools or tremors. An electrocardiogram showed sinus rhythm with a rate of 72 beats per minute. So our patient had subclinical thyrotoxicosis without overt clinical symptoms and signs.

He was started on a low-dose steroid: prednisolone 10 mg daily. He was feeling well and fever free on third day after starting steroids and was discharged. Low-dose prednisolone was continued for 1 week and stopped. As he was asymptomatic, he was not started on NSAIDs. He was reviewed in 1 month. He was asymptomatic and his thyroid profile was normal with TSH of 2.5 MIU/mL and free thyroxine and free triiodothyronine in the lower normal range. A follow-up was arranged to review his thyroid profile in 6 months.

## Discussion

PUO, first described by Petersdorf and Beeson, is among the most difficult diagnostic problems encountered in internal medicine [1]. PUO was defined as temperatures of > 38.3 °C (> 101 °F) on several occasions, duration of fever of > 3 weeks, and failure to reach a diagnosis despite 1 week of in-patient investigation [1]. Infections, malignancies, and autoimmune conditions make the bulk of etiopathogenesis of PUO. Endocrine disorders as a cause of PUO are rare. We describe a case of PUO due to subacute thyroiditis.

De Quervain’s thyroiditis (also termed giant cell or granulomatous thyroiditis) is a subacute inflammation of the thyroid, which accounts for 5% of thyroid disorders [2]. It usually follows viral infection commonly an upper respiratory tract infection and spontaneously regresses [3, 4]. Common viruses implicated in the etiopathogenesis include mumps, echovirus, hepatitis b, hepatitis C, cytomegalovirus, and coxsackieviruses [5]. Some genetic factors, such as human leukocyte antigen (HLA) Bw35, might affect individuals’ susceptibility to possible viral pathogens; they were reported to play a role in the pathogenesis of the disease [6].

Clinically subacute thyroiditis is associated with severe pain that is usually localized to the anterior aspect of the neck and may radiate up to the jaw or ear. The patient has tenderness of the thyroid gland on palpation and small diffuse goiters are frequently present. Patients commonly experience fatigue and mild thyrotoxic symptoms. Low grade fever is reported in a smaller percentage [7]. Interestingly our patient presented with fever with generalized malaise without neck pain and thyrotoxic symptoms. Although the literature describes an exquisitely painful thyroid, our patient had mild tenderness which he revealed only on questioning. He did not have clinically palpable thyroid tissue, but cervical lymphadenopathy. Cervical lymphadenopathy is not commonly reported with subacute thyroiditis. Solivetti *et al*. described preliminary results of a study on the importance of juxtajugular and supra-isthmus lymph node enlargement in the USA as a sign of occult subacute thyroiditis in which 91% of all patients with thyroiditis had supra-isthmus or juxtajugular lymph node enlargement [8]. Rarely, the main presentation of subacute thyroiditis could be fever which may go undiagnosed for weeks [9, 10].

Raised ESR is a common finding in subacute thyroiditis [11]. High CRP can also be seen in subacute thyroiditis [12]. Our patient had both high ESR and CRP. In subacute thyroiditis most patients have negative antibodies as seen in our patient. Erdem *et al*. reported positive antithyroglobulin antibodies among 20% of patients and thyroid peroxidase antibodies among 4% patients with subacute thyroiditis [13].

There is no universal guideline on management of subacute thyroiditis. Various management approaches reported in the literature for subacute thyroiditis indicate that some patients require no treatment, while many others frequently require NSAIDs as symptomatic analgesic and anti-inflammatory agents. If the symptoms persist despite the use of an NSAID or in cases with severe symptoms, corticosteroids are usually prescribed only after excluding acute suppurative thyroiditis [14]. Fatourechi *et al*. described that prescribing corticosteroids did not protect against late occurrence of hypothyroidism and interestingly long-term hypothyroidism was significantly more common in the group who received corticosteroid therapy [15]. There is no consensus regarding the dose of corticosteroids in the treatment of subacute thyroiditis. Low-dose steroids (dose of 10 mg of prednisolone) have been used in many studies [16, 17]. Our patient responded well to a low-dose steroid at a dose of 10 mg of prednisolone. Although there is no consensus recommendation for regular screening for hypothyroidism after an episode of subacute thyroiditis, many studies have found the occurrence of subclinical or overt hypothyroidism on long-term follow-up. Das described seven out of twelve patients developing subclinical or overt hypothyroidism at 3 months of follow-up [18]. Permanent hypothyroidism was documented in 14.3% of patients in a case series of 25 patients by Alfadda *et al*. [7]. In Olmsted County, Minnesota, USA, a study by Fatourechi *et al*. described early hypothyroidism (within first 6 to 12 months) in 34% and late hypothyroidism (after 1 year) in 15% of the study cohort of 94 patients [15]. So patients recovering from subacute hypothyroidism need regular short-term and long-term follow-up with regard to their thyroid status to look for the occurrence of early or late hypothyroidism.

## Conclusions

Although endocrine disorders as a cause of PUO are rare, subacute thyroiditis should be considered in the differential diagnosis even if clinical symptoms of thyrotoxicosis are not present. In the absence of clinical signs and symptoms, an abnormal thyroid function test will lead to a diagnosis of subacute thyroiditis in a case of PUO and may prevent the patient being subjected to many unnecessary investigations in the diagnostic workup of PUO.
